# Women’s understanding of economic abuse in North-Western Tanzania

**DOI:** 10.1177/17455065211042180

**Published:** 2021-09-08

**Authors:** Anna de Serpa Pimentel, Gerry Mshana, Diana Aloyce, Esther Peter, Zaina Mchome, Donati Malibwa, Annapoorna Dwarumpudi, Saidi Kapiga, Heidi Stöckl

**Affiliations:** 1 Hull York Medical School, Hull, UK; 2 Mwanza Intervention Trials Unit, Mwanza, Tanzania; 3 Gender Violence and Health Centre, Department of Global Health and Development, London School of Hygiene & Tropical Medicine, London, UK; 4 Department of Infectious Disease Epidemiology, London School of Hygiene & Tropical Medicine, London, UK; 5 Institute for Medical Information Processing, Biometry and Epidemiology, Ludwig-Maximilians-Universität München, München, Germany

**Keywords:** economic abuse, gender-based violence, intimate partner violence

## Abstract

**Introduction:**

Economic abuse is a form of intimate partner violence that still lacks a clear conceptualization and therefore is often overlooked next to physical, sexual and psychological abuse. While existing categorizations recognize economic intimate partner violence as economic control, economic exploitation and employment sabotage, current measurements of economic abuse rarely capture all its forms, and the issue has not been widely explored in low- and middle-income country settings.

**Methods:**

We conducted in-depth interviews with 18 women in Mwanza, Tanzania to understand local perceptions and experiences of economic intimate partner violence. We used a thematic analysis approach.

**Results:**

Our study illustrates the complexity of economic abuse as a unique form of intimate partner violence, with women experiencing economic exploitation, employment sabotage, economic control and male economic irresponsibility. Gender norms and expectations actively played a key role in furthering abusive economic behaviour as women attempted to generate their own income and participate in financial decisions. Women’s constructs and reactions to economic abuse diverged sharply from the traditional marital expectations of dutifully accepting male control and the men being the main breadwinners in the family. Despite it being widespread, women did not find economic abuse acceptable.

**Conclusion:**

The results highlight that economic abuse is a complex issue and that more research on the pathways and manifestations of economic abuse globally would be beneficial. Existing measurement tools should be widened to address all dimensions of economic abuse. Addressing economic abuse will require multi-strategy interventions, working at the individual and community-level to address gender roles and masculinity norms, working with both men and women.

## Introduction

Violence against women is pervasive globally, with intimate partner violence (IPV) causing physical, sexual and psychological harm.^1^ Recent estimates suggest that one-third of women globally have experienced physical and/or sexual violence by a partner or non-partner sexual violence in their lifetime.^2^

Despite emerging evidence of economic violence as a sub-form of IPV, there is a lack of attention and focus on this form of violence in comparison to physical or sexual violence and it is not typically acknowledged as a unique form of power and control.^3^ Economic abuse is often captured as a form of psychological abuse or controlling behaviour or tactics in survey instruments that force financial dependence and social isolation, impacting mental and physical health.^4,5^ Economic abuse can include ‘behaviours that control a woman’s ability to acquire, use, and maintain economic resources, thus threatening her economic security and potential for self-sufficiency’.^6^ Behaviours include preventing or limiting time at work or school, stealing money, harassment at work or school, creating debt, preventing access to money or completely controlling financial decisions.^6^

Economic insecurity and abuse is often simplified to be a consequence of IPV, leading to poverty, financial risk and financial insecurity.^7^ Due to the myriad and culturally diverse ways economic abuse can manifest, there is a general lack of consensus on what fully constitutes economic IPV and the best practices to individually measure these constructs.^8^

Among the women experiencing IPV, studies have found high rates of economic abuse, suggesting that this subtle and common form of violence is highly prevalent.^8^ For example, in South Africa, 45% of women experienced a combination of emotional and economic IPV. The women reported both forms of IPV had increased symptoms of depression, with economic IPV playing a significant role in triggering suicidal ideation.^9^ Socio-economic consequences of economic IPV include increased dependency on the abusive partner for food, resources, housing, child care and transport.^10^ Systematic reviews on economic empowerment in low- and middle-income countries (LMIC) also illustrates that women with fewer economic resources are less able to leave their partner or negotiate change, leading to higher endurance for IPV.^11,12^ Economic violence is a multi-faceted issue that is informed by socio-economic, cultural and gendered factors and that it is situated in a complex relationship between income, control and gendered power. However, existing findings largely focus on Western populations and are not be applicable to women in LMIC.

A multi-country review on definitions and measures of economic abuse found that studies tended to conceptualize economic abuse around three distinct strategies: economic control, economic exploitation and employment sabotage – as summarized in Table 1.^13^ Other literature also identifies ‘Refusal to contribute’ as a form of economic abuse which includes not being accountable for spending, refusing to work, refusing to pay bills and refusing to contribute to the costs of raising children.^8,14^ Globally, there is no agreed upon index to measure economic abuse. The most prominent scale of economic abuse (SEA) contains two subscales on Economic Exploitation (11 items) and Economic Control (17 items).^6^ A shortened instrument to measure economic abuse, the SEA-12 has three subscales: Economic Control, Employment Sabotage and Economic Exploitation.^8,13^ Furthermore, both scales have only been tested in the United States and their validity in LMIC contexts has not yet been explored.^3,5^

**Table 1.** Economic abuse definitions and tactics.

Type of economic abuse	Definition	Tactics
Economic control	Economic control occurs when the abuser prevents the woman from having access to or knowledge of the finances and from having any financial decision making power.	• Controlling financial resources • Denying basic necessities • Tracking the use of money • Withholding or hiding jointly earned money • Lying about shared property and assets • Refusing access to a bank account.
Employment sabotage	Employment sabotage encompasses behaviours that prevent the woman from obtaining or maintaining employment.	• Forbidding, discouraging, actively interfering with employment or educational endeavours • Harassing them at their place of employment • Obstructing them from receiving other forms of income such as child support, public assistance or disability payment
Economic exploitation	Economic exploitation occurs when the abuser intentionally engages in behaviours aimed to destroying the woman’s financial resources or credit.	• Stealing money, checks, ATM card • Opening a line of credit under their partner’s name • Gambling jointly earned money

ATM: automated teller machine.

Describes Economic control, Employment Sabotage and Economic Exploitation based on Stylianou (2018).

It may be difficult to conceptualize economic abuse holistically, especially in LMIC where couples are under significant financial hardship, as the distinction between economically abusive patterns and economic instability may be blurred. It is therefore possible that economic abuse may be under-studied as a form of IPV throughout the relationship, or men’s inability to provide due to financial hardship may be perceived as violent. Furthermore, economic relationships may be financially unequal yet be mutually agreed upon.^14^ To further expand our understanding of and to inform existing definitions of economic abuse, we investigated women’s experiences, manifestations and perceptions of economic abuse in North-West Tanzania.

## Methods

### Study setting

The study took place in Mwanza city, a peri-urban area in North Western Tanzania. Mwanza is Tanzania’s second largest city, located at the Southern shores of Lake Victoria. In 2012, Mwanza had a population of 722,592,^15^ with an estimated GDP/capita in 2016 of US$ 13,748 (9.7%), compared to US$ 24,129 (17%) in Dar es Salaam.^16^ The city is a major business and commercial trade hub for neighbouring countries of Kenya, Uganda, Burundi and Rwanda and the lake Victoria region itself.^17^ As of 2015, 30.4% of women had no formal or completed primary education, 46% completed primary and 23.4% had higher education, with 67% being currently in employment compared to 87% of men.^18^ The majority of women worked in the agriculture setting (52%), followed by unskilled manual work (23%).^18^ In Tanzania, 42% of married women reported having experienced physical or sexual IPV.^18^ In a study in Mwanza measuring the prevalence of IPV among women, it was found that about 61% of women reported ever experiencing physical and/or sexual IPV and 34% reported economic abuse during the past 12 months; with 96% of respondents earning an income and 28% contributing more financially to the household than their partner.^19^ While women’s income was protective against IPV, women who contributed more financially than their partners had greater IPV risk; with poverty and tensions over men’s inability to provide emerging as potentially important drivers of this association.^20^

### Study design and data collection

This study is based on 18 in-depth interviews with women in Mwanza, North-Western Tanzania, carried out between May and July 2019, as part of the MAISHA (Kiswahili for Life) longitudinal study. The MAISHA longitudinal study is based on the MAISHA trials, IPV intervention trials combining participatory gender and violence training with an on-going microfinance intervention for women.^21^ Women, participants of women in the control group of the MAISHA trials, were included into the study if they had reported changes in their experience of sexual IPV between the baseline and endline MAISHA trials (see Table 2 for participants’ characteristics). The recruitment of new participants stopped after the 18 interviews as data saturation was met as no new information related to the topics of interest was coming from the interviews.

**Table 2.** Participant characteristics.

Participant ID	Age (years)	Marital status	#Children < 18 years	Level of education	Occupation	Religion	Tribe
IDI-#01	43	Married	4	Secondary	Tailor	Christian	Ngoni
IDI-#02	45	Married	5	Primary	Farmer	Christian	Sukuma
IDI-#03	44	Married	2	Secondary	Farmer	Adventist	Sukuma
IDI-#04	48	Widow	3	Primary	Entrepreneur	Muslim	Haya
IDI-#05	43	Married	1	Secondary	Unemployed	Muslim	Pare
IDI-#06	32	Divorced	2	Primary	Unemployed	Christian	Jita
IDI-#05	37	Married	3	Primary	Entrepreneur	Christian	Nyakyusa
IDI-#08	27	Single	1	Diploma^a^	Entrepreneur	Christian	Sukuma
IDI-#09	45	Married	1	Primary	Entrepreneur	Christian	Ngoni
IDI-#10	37	Divorced	4	Primary	Unemployed	Christian	Sukuma
IDI-#11	45	Married	None	Primary	Entrepreneur	Christian	Haya
IDI-#12	30	Married	2	Primary	Entrepreneur	Christian	Nyambo
IDI-#13	57	Married	1	Primary	Entrepreneur	Christian	Sukuma
IDI-#14	36	Divorce	3	Diploma^a^	Hotelier	Christian	Sukuma
IDI-#15	43	Married	2	Primary	Entrepreneur	Christian	Angaza
IDI-#16	41	Married	2	Primary	Entrepreneur	Muslim	Haya
IDI-#17	49	Married	1	Primary	Unemployed	Christian	Sukuma
IDI-#18	43	Married	2	Primary	Entrepreneur	Muslim	Sukuma

a A 1- to 2-year programme offered after secondary education focusing on a specific skill or field.

The interviews were conducted by two Tanzanian female interviewers (D.A. and E.P.), aged 26 and 27 years, each conducting nine interviews, trained on qualitative interviewing techniques, gender issues, violence and ethical issues related to IPV. After women gave written informed consent, all interviews were audio-recorded and took place in a location chosen by the participant for their comfort, the majority in the participants’ homes. Special care was taken that the woman could be interviewed on her own and interviews were interrupted once another person, especially their partner, could overhear the interviews. Piloted topic guides with open-ended questions and probes were used to explore how women conceptualized economic abuse (see Supplementary File). Questions asked in general how women believed an ideal couple deals with finances, how much control partners should have over each other’s finances, probing for both partners over each other, what kind of expenses a woman can expect her partner to pay and vice versa, what kind of behaviour regarding a couple’s finances is normal and what kind of behaviour is considered inacceptable followed by what behaviour they consider to constitute economic abuse.

Given the sensitivity of the topic, open questions were used to establish rapport and initiate the discussion. The interviews lasted between one to two hours. Detailed notes were collected throughout the interviews which created the field notes to document anything of interest such as emotions or disturbances during the interviews. The interviews were conducted face-to-face in Swahili, transcribed verbatim and later translated to English. A sample of the transcripts were translated back into Swahili by a different translator to evaluate the quality of the translations.

## Analysis

The 18 interviews were analysed using thematic analysis because it offered a theoretically flexible approach in contrast to theoretically bounded approaches such as grounded theory and narrative analysis.^22^ This was appropriate as economic abuse is a relatively early area of research, theory around economic abuse is not yet well established.^6^ The main author first read all full transcripts carefully to familiarize herself with the data and to identify initial codes relevant to the research topic. Those inductively generated codes that emerged from the data itself were coded using NVivo 12 software. In a second step, with the guidance of the literature review, especially the definitions of economic abuse in Table 1, the coding frame was revisited to check compliance with existing definitions of economic abuse. Given the substantive overlap, the codes were re-modelled to reflect the experience of the participants with economic abuse. To check the codes were correct, data in the codes were re-read line by line. Thereafter, patterns and relationships between the codes were identified, resulting in four main themes, reflecting women’s experiences of economic abuse (Braun & Clarke 2006). The coding was verified by comparing it to previous coding completed by E.P. and D.A. for a different analysis, two graduate researchers who have also conducted the interviews. Discrepancies were resolved through discussion by the authors. The authors also discussed which quotations were coherent and reflective of the themes that had been identified and confirmed throughout the process of analysis.^23^ The analysis also took account of the field notes that recorded the overall mood of the interviews, details of the women’s demeanours and non-verbal cues.

Ethics approval for this qualitative study was obtained from the Ethics Committee of the London School of Hygiene and Tropical Medicine (Ref: 11918-3); and the National Health Research Ethics Committee (NatHREC) in Tanzania (NIMR/HQ/R.8a/Vol.1x12475). The research followed the ethical recommendations on researching gender-based violence,^24^ ensuring that participants are aware that their participation is voluntary, information will be related confidentially, interviewers are trained on violence against women and girls research, the interview takes place in a private setting and women receive referral options if needed. Everyone who participated received 8000 Tanzanian shillings (US$ 3.45) after the interview as a reimbursement for their time and expenses.

## Results

Most of the interviewed women (17 out of 18) had experienced economic abuse at some point in their lifetime from a current or previous intimate partner. Four broad themes emerged from the women’s accounts of economic abuse: (1) economic exploitation, (2) employment sabotage, (3) economic control and (4) male economic irresponsibility.

### Economic exploitation

Women in this study who experienced economic exploitation from their partners, described their partners taking the money intended for the family without consent, building up debt under their names and not paying bills listed under both of their names. Women also shared stories of being coerced or forced to lend their partners money or take out loans, with their partners not paying back this amount. This type of economic exploitation hindered the financial mobility of these women and caused further instability especially for women in the study who were entrepreneurs. Overall, the participants conceptualized a man taking their money as economic abuse, citing the importance of their hard work and their future plans for the money. One participant highlighted the discrepancy between the socio-economic expectations of men as the main providers for women and the stark realities that women experience:

My partner would take the money I earned, though not by force. He would tell me that he need two or three millions. He would say, ‘I will pay you back after one week’. And he doesn’t do that. Once I give him [the money], it’s gone. When I ask for my money back, he will act as if he doesn’t understand me. (44, married, entrepreneur)

Men taking money were more likely to lie to their partners or deny their actions, placing women in uncomfortable social and economic positions:

I lost the money inside the house. I thought that it cannot be a child who took it, this is my partner. I lost about one million and six hundred thousand shillings . . . So, something like that I was not pleased with in general. I told him, I was not pleased . . . A child cannot find the money considering where I kept the money. (She argued with her husband) ‘So it is me who took the money’?! ‘It is you, where have you taken it to?’ ‘I didn’t take it’. ‘just tell me where have you taken it to?’. ‘I didn’t take the money’. (45, married, farmer)

While some women took loans in secret and /or utilized precautions against stealing as tactics to protect themselves from economic abuse, some confronted their partners verbally to defend their actions:

Even when I decide to take a loan, I do it in secret . . . I invest that money on my own . . . I do as I please as he awaits. He later asks . . . ‘why haven’t you taken the loan’. I tell him (lie) that the BRAC officer is unavailable. He eventually realizes that ‘this woman does not want to do this’ and leaves me to my devices. (44, married, entrepreneur)

Women also reported tactics their partners utilized to evade economic responsibilities, such as leaving the house when the rent is due, placing the responsibility on the woman, basically living off the woman’s money:

For instance, we are living together and you know that at a certain date we have to pay for rent . . . a man refuses to pay for rent and he does not spend a day at home. When the owner of the house comes, he finds a woman . . . he then starts shouting as he needs his rent. It is violence. (32, divorced, unemployed)

Women also perceived it as economic abuse if their partner asked them to lend him money but did not pay it back. Similarly, if their partners force them to give them money they have worked hard for and planned to spend for their own or their family’s needs:

It’s violence because you have toiled and sweated for this thing. A person comes and tells you that he wants to borrow that thing for a week while you have your own plans. You refuse by telling him that . . . my friend, I have my own plans . . . I want to spend this money on my own activities . . . on my house . . . to do something . . . I need this money. (He would say) ‘Just borrow me the money; I will pay you back in a week’. But once you give him that money, he is gone (claps once to emphasize the word ‘gone’) You kiss your money good-bye. You will never set your eyes on that money again. (44, married, entrepreneur)

Some women also perceived that traditional gender roles can create a power imbalance that facilitates economic exploitation. In this example, a woman acknowledges the role of consent and coercion in economic abuse and illustrates how gender perceptions justify financial exploitation:

Even if you are in need, and I have that money, there is a way to talk and ask me for that certain amount and I will give you if I have it, instead of taking it by force, that will be violence. You take it by force just because I am a woman and you are a man. You know I can’t beat you up. It would be a patriarchal system where a man would just say: I am the head/man of this house, I can do anything I want without being answerable to anyone. (49, married, unemployed)

### Sabotage of employment and income generation activities

Some women experienced economic abuse in the form of their partner prohibiting or disrupting them from earning money or gaining employment. This experience was observed at varying extremes with some partners disapproving and some actively preventing women from income generating activities. All participants recognized employment sabotage as a form of economic abuse. Women theorized different reasons for their partners’ actions, such as protecting her from the advances of other men, keeping her at home to look after the children, keeping her financially dependent, not letting her see where he works, to keep up his image and not wanting her out of his sight. The latent reason underlying all of these reasons is maintaining and securing the control that a man has over his partner. Some narratives also pointed to societal pressure and gender norms as factors contributing to employment sabotage:

I: Did you ever ask why he wanted you to do nothing?

R: Yes, he said I was shaming him.

I: How did he say you were shaming him?

R: He said that people will think he has bad character.

I: Which bad character did he speak of?

R: If I was fetching water, it shames him, because it will seem like he is not taking care of me. This was true; he wasn’t taking care of me

(37, divorced, unemployed)

He agreed at the beginning and I started the process but later on he came to realise that I will build this house on my own. (He thinks:) A woman can’t build this house. I told him to stop having those thoughts. If you are thinking that way we will never succeed. (37, married, entrepreneur)

The women also outlined the various tactics their partners used to purposefully prevent them from working:

He could follow you up to the place you are working, forces to take your things or do anything. [. . .] He would wait for you at home. Let’s say you have brought things/commodities for trading with. He could pour kerosene on it so they could be destroyed, for you not to sell them [. . .] And this happened many times. (37, divorced, unemployed)

The women conceptualized employment sabotage as a form of control and symbol of power from the man over his partner. Furthermore, the study participants perceived that men regarded women as needing protection, unable to handle the working environment, or earning money:

R: Yes, he prohibits you from working, and my husband used to prohibit me from doing anything.

I: Why would he prohibit you from doing any work?

R: Just to want you to be below him and dependent on him for everything. After getting money, they think women change when it is not true for it depends on individual minds.

(41, married, entrepreneur)

Women in this study asserted their right to work and their desire to contribute to their families’ economic status. This inclination to engage in income-generating activities contributed to the perception of employment sabotage as a form of economic IPV:

You may find that a woman may do a business, or wishes to do a business. Mm. But a man may prevent her from doing so, he just want a woman to stay and not to work. [. . .] That is violence as well because she will have her own needs, she would want certain things and when she tells you, you will just say that you do not have money, but if she worked, she could afford it by herself, or maybe she may wish to help her family on certain matters. (37, married, entrepreneur)

### Economic control

Women experienced economic control from their partners, resulting in a diminished role in the economic decision-making process. Some participants reported that their partners tracked the use of money, controlled and limited access to financial resources and denied access to necessities. Women perceived these actions as violence, citing the emotional stress and diminishment caused by their partners. Some women also asserted that open communication and joint financial planning was essential for a good relationship:

He didn’t show me! He didn’t!! To the extent that I don’t even know where the bank card is!! He is the only one who knows. (30, married, entrepreneur)

He tells you that I have a project somewhere but if you tell him to elaborate more he doesn’t, he is a very secretive man. He doesn’t tell you as his wife that I have this and this. Maybe I have a farm with someone, I work. What kind of work is that, . . . besides that one which he was doing of CD, he doesn’t tell you his other works, he doesn’t say what kind of work does he share, what capital does he get or how much is he paid. (27, single, entrepreneur)

As seen in instances of economic exploitation, women continued to illustrate how socio-cultural ideals disregard women from financial conversations:

I do not feel good. I like it when he involves me, but he does not do that. He says that men’s issues are for men, a woman is not a person to share such money issues with. (37, married, entrepreneur)

Some women stated that their partners tracked their spending and monitored their finances, showcasing the inequality in financial oversight and the resulting emotional stress of these actions:

He is still in this habit of buying food on his own. If he gives me money to go and buy food, he will calculate on his own. For example, if I need thirty kilograms of rice, he will leave me with the exact money depending on the price of what I want to buy as he knows it. He wants it to be exactly the same, if it is less even by a thousand shillings, I will be abused as a lizard. That’s the life am currently living. (37, married, entrepreneur)

I don’t know how much he earns, I don’t know where his money is, and I even don’t know how he wants to spend his money. But when I have earned money from a certain activity, he will want to know how I will spend that money. He might even tell me to use the money I have earned on a certain thing he wants. It is something that hurts me a lot. (49, married, unemployed)

When her partner did not provide basic necessities for the family even though he could, a participant acknowledged this neglect as a tactic for maintaining control and limiting her independence. The following quote illustrates a woman perceiving economic control as a deliberate and intentional form of violence:

A man uses money to abuse or oppress you. Because he thinks if he doesn’t provide you with money, you can’t dress your hair and look good, you can’t wear good clothes, you can’t put your family in a good condition, can’t eat well so he feels like you will just be there like something. He doesn’t want to see you looking good because his mind tells him you will be seen by other men if you look good. So, he uses that situation of not providing you with money as a way to protect you. (45, married, entrepreneur)

### Male economic irresponsibility

Women in this study reported that they were forced to take charge of providing for their family while their partner did not contribute or did not contribute sufficiently. Many women provided for their children’s school fees and food because their partner was either absent or did not contribute. Women generally conceptualized this situation as abusive because it left the household without resources. The participants also believed that the man should be breadwinner and wives should only be expected to make small financial contributions to the household. This was especially the case when their partners spent the household money on alcohol, a mistress or refused to find a job, resulting in a lack of resources for the household. In several cases, women experienced long periods of time with no financial support from their partners:

You see, my husband stayed at home for more than 3 years without a job, without anything. Honestly, in those situations, one person in a relationship becomes affected psychologically. (speaking hesitantly) You see? He didn’t have a job and spent most of his time sleeping. (43, married, entrepreneur)

When their partners did not provide financial support for the family, women took over the responsibility of household expenses and school fees to prevent economic instability:

He tells me, ‘I don’t have money’. Now as a mother of the family, I don’t know what will happen tomorrow. . . . if a mother can support the children with her own income (she should do that). If a mother just sleeps at home, children may end up on the streets, smoking marijuana. Many fathers nowadays shun their responsibilities. A lot of men! ‘I don’t have money right now. Ask your mother’. (43, married, entrepreneur)

Your friend might tell you . . . ‘my husband is not working . . . he is counting on me to do this job so that he and family can eat. There are many people like that’. It’s true. You might find this woman who works until 01:00 am in the night waiting on customers. . . . to get cash in order to meet her family needs whilst the husband is just sitting there doing nothing. (44, married, entrepreneur)

There was an emphasis from women that their partners spent the money frivolously, quickly and irresponsibly. Women conceptualized this as violence because of the lack of resources this left in the home:

Even when you gave him two or three million shillings, (he would squander the money) in less than a week. I mentioned that in the beginning. A week was too long a time (to still have some money left). He would tell you he doesn’t have even a single cent. Doesn’t have any money. And you don’t see what he has done with the money. (44, married, entrepreneur)

There is one which I see to me maybe we can say you find sometimes my husband drinks alcohol. It happens that he comes back at home drunk but here at home even money for buying sardines is missing, that is mistreatment! If I ask you for money for buying sardines and it is not found, I will not understand. (45, married, farmer)

Some women reported that their partners misused limited family resources by spending money on their mistress, rather than on their family and household expenses. The participants reported that they had to take more control over economic roles to support the family since the partner was neglecting his responsibilities as the primary provider:

My husband was okay taking care of his mistress child instead of taking care of his own child. He does not provide us with proper food at home, but when it comes to his mistress, he was buying proper foods and even dare to show off when he goes to her house. It hurt me alot and that’s why I couldn’t bear the relationship. (43, married, entrepreneur)

Some women had to borrow money, relying on other women, their families or neighbours to support their families. Women conceptualized this occurrence as abuse and asserted traditional gender roles as the expected norm in a financial relationship:

You struggle on your own. Mm, I do my own business I get some money, I borrow from my neighbours like one or two hundred thousand you pay later on, you invest in financial groups you generate then later on you pay them back. (43, married, entrepreneur)

You are the one to help him instead of him helping you, that is mistreatment. Yes, a man must hustle and look for money to help his partner and not the woman hustling to help the husband. (41, married, entrepreneur)

Some women perceived having to take on the burden of responsibility as an abusive situation because it is forced upon them and because of the emotional stress it places on them:

It is like someone is forcing you as he puts all the burden of his responsibilities on you. You face it. And now, as he puts the burden on you and you may be having a lot on in your head. In the end it even affects your performance in your life activities. When you have so much thoughts like where to get school fees from, this and that, that is mistreatment. (36, divorced, hotelier)

## Discussion

**Figure 1.**
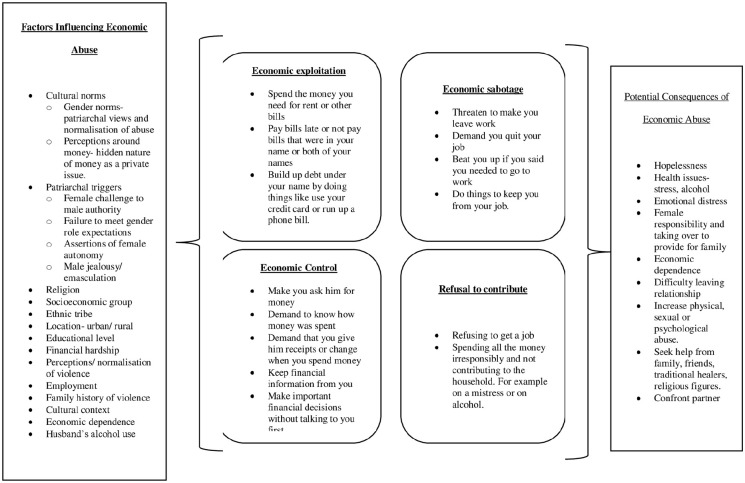
Conceptual framework of economic abuse.

Findings from this study provide a multi-layered approach to recognize and understand economic IPV and identified four main forms of economic violence experienced by women in North-West Tanzania: economic exploitation, employment sabotage, economic control and male economic irresponsibility. These four concepts of economic violence observed in this study expand the recent definitions of economic IPV as partially outlined in Table 1 and inform established pathways of abuse.^8,24^ There was an intriguing tension between the first three concepts of economic control, sabotage and exploitation and the later concept of male economic irresponsibility, with the first three depicting situations that are influenced by structural constraints women face in their ability to take up economic responsibilities and be involved in decision making, often through existing social norms. The concept of male economic irresponsibility compared to that refers to economic abuse that occurs in relationships were women believe their partner is not living up to expected social norms and in which they are pressured into accepting economic responsibilities and roles that would expect their partners to be responsible for. In both cases, social norms around what economic roles men and women are able to play but are also expected to play in a relationship can led constrains facilitating economic abuse or lead to broken expectations based on social norms that women perceive as economically abusive. The terminology ‘Male economic irresponsibility’ was used in place of ‘Refusal to contribute’ that was used in prior studies as it reflected the indifferent attitudes portrayed by men through the women’s lenses. This study also demonstrates additional ways in which economic IPV is inflicted on women – not just by refusing to contribute money they might have but also by not actively looking for employment, spending the money they had on mistresses and alcohol or gambling and finding it acceptable that women have to take loans or ask relatives for money instead of providing for them. The findings illustrate the multi-faceted nature of economic abuse, the gender norms that facilitate violence, and the challenges women face in maintaining economic mobility and supporting their families. The potential consequences of economic abuse but also its co-occurrence with emotional abuse established in this study also show how closely linked economic abuse can be with emotional IPV and that it can be difficult to disentangle the two. This is especially the case when social norms about what roles women and men should play in relationships come into play. The new insights generated in this study have been summarized in a conceptual framework (Figure 1) that also capitalizes on the conceptualization by Postmus et al.^13^ by adding ‘Refusal to Contribute’ as one of the main forms of economic abuse. It further highlights several of the complexities and influencing factors around economic abuse that need to be considered.

Despite the patriarchal attitudes in Tanzania, the women in this study asserted clear recognition of actions pertaining economic abuse and provided evidence for economic abuse as a dimension of IPV.^18^ Women’s constructs and reactions to economic abuse diverged sharply from the traditional marital expectations of dutifully accepting male control and the men being the main breadwinners in the family.^25^ While economic abuse is not well established as a form of violence, it was rarely considered normal or acceptable by participants. The women provided clear reasoning as to why certain actions were abusive, citing violations of trust, respect, independence and familial responsibility. Women in this study did not accept economic abuse as something natural or as a cultural norm. On the contrary, women worked hard to combat abuse through many resourceful strategies. Reinforced by current literature, four main defensive strategies were employed by the women in this study, including hiding money; negotiating with their abuser; seeking help from family and friends; and pursuing employment and building social networks for help with economic goals.^26^ In this study women actively participated in household purchases, providing basic household needs, built houses, provide for children’s education and took care of themselves, despite male economic irresponsibility. Women generally conceptualized the situation they were in as abusive because their partner did not fulfil the expected role as provider and put the burden on them instead. They were more vocal about economic abuse if they cared for minor children and either needed their own or their partners income to meet their children’s need for food, medical bills and educational expenses. This finding supports situating future research on the conceptualization of economic abuse within gender relations and cultural context.

In this study, challenges to men’s traditional gender role often triggered instances of economic abuse. Being female was often perceived as the reason for employment sabotage as well as a reason why women should not have control over finances. This is reflected in the hierarchical dimension of gender beliefs that men are viewed as more status worthy and competent at the activities that ‘count the most’, whereas women are seen as better at less valued, communal tasks.^27^ In a study in Cote d’Ivoire, women who pursued economic opportunities were seen as a threat by their partners, and this perceived loss of control and disregard for traditional gender roles were undying causes of all forms of IPV experienced by women.^28^ When men cannot adequately provide for basic household needs because of economic difficulties, women’s active economic engagement may trigger abuse as the man might feel emasculated.^29^ Tensions over men’s refusal to provide and feelings of emasculation emerged as potentially important drivers of economic IPV among women.^30^

‘Refusal to contribute’ or Male economic irresponsibility (as indicated in this study) is not considered economic abuse consistently in the literature.^8^ This pathway is often viewed as a passive form of abuse or neglect rather than actively controlling, sabotaging or using tactics against a woman. However, women in our study perceived ‘Male economic irresponsibility’ as violence, suggesting that situational factors may affect this conceptualization. For example, a man not providing may be considered violent in this particular context, where, according to national statics women are not as equally able to provide for their family – in 2016, 42% of women versus 10% of men in Tanzania were not paid for their work.^18^

This study furthermore provided strong evidence that measurement tools in surveys capturing economic abuse should be expanded to consider the theme of male economic irresponsibility in correlation with employment sabotage, economic exploitation, economic control, after validating them. For the above-mentioned questions on economic abuse in the MAISHA study, additional questions should be included on ‘has your partner ever taken money away from you that you earnt, has your partner built up debt under your name or has he refused to give money for household expenses, even when he has money for other things?’

There are several limitations that need to be considered when interpreting the findings of this study. This study was based on 18 in-depth interviews in an urban city in North-West Tanzania and therefore cannot be easily generalized to the rest of Tanzania or elsewhere. IPV is a sensitive topic, and interviewees might have also given socially desirable response to some of the question or refrained from talking openly about violence. Additional interviews with a concentration on economic abuse would increase the depth and understanding of the complexities and the perspectives on this form of violence. Participatory research would further contribute to the existing knowledge as it would allow the women to be actively involved in the research process and reflect on their experiences in their own voices. In addition to this, ethnographic research would potentially provide a more holistic understanding of economic abuse in this specific context. Future studies should incorporate a larger sample size that includes a wider range of ethnic groups, education levels, occupations, and urban and rural populations. There is also a clear need to investigate the male perspective on economic abuse, both in terms of perpetration as well as experience, as husband and wife claims to decision-making authority have found to vary significantly, depending on the type of decision they needed to make.^31^ There was also no clear measure of the women’s socioeconomic status other than occupation, which impeded an assessment on income and economic empowerment.

## Conclusion

This study adds to the growing body of evidence around economic abuse as a unique form of IPV. The findings of the study suggest that economic abuse is common in Mwanza and emphasizes the continued need to understand economic abuse globally and in Tanzania. Women’s perceptions of economic abuse were broad, and they had clear understanding of economic abuse and its overwhelming impact on the quality of their lives. The role of gender was particularly significant in women’s perceptions of violence and challenging gender norms was often an underlying cause for further IPV. More research into understanding economic abuse in different contexts, especially financial hardships and from the male perspective may affect the conceptualization of economic abuse and expand current definitions and measurements. The results highlight that economic abuse is a complex issue, consequently multi-strategy interventions are recommended, such as interventions that empower women, and working with both men and women, within couples and at the community-level to address gender roles and masculinity norms. The finding of this study are likely to be helpful in tailoring these interventions to promote equity between women and men, provide economic opportunities for women, inform them of their rights, reach out to men and change societal beliefs and attitudes that permit exploitative behaviour. Economic education programmes need to be considered as a critical intervention for women experiencing IPV because they have the potential to increase their economic self-efficacy, financial literacy and financial behaviours, as well as build their capacity to address the economic consequences of economic abuse.

## Supplemental Material

sj-docx-1-whe-10.1177_17455065211042180 – Supplemental material for Women’s understanding of economic abuse in North-Western Tanzania Click here for additional data file. Supplemental material, sj-docx-1-whe-10.1177_17455065211042180 for Women’s understanding of economic abuse in North-Western Tanzania by Anna de Serpa Pimentel, Gerry Mshana, Diana Aloyce, Esther Peter, Zaina Mchome, Donati Malibwa, Annapoorna Dwarumpudi, Saidi Kapiga and Heidi Stöckl in Women’s Health

sj-docx-2-whe-10.1177_17455065211042180 – Supplemental material for Women’s understanding of economic abuse in North-Western Tanzania Click here for additional data file. Supplemental material, sj-docx-2-whe-10.1177_17455065211042180 for Women’s understanding of economic abuse in North-Western Tanzania by Anna de Serpa Pimentel, Gerry Mshana, Diana Aloyce, Esther Peter, Zaina Mchome, Donati Malibwa, Annapoorna Dwarumpudi, Saidi Kapiga and Heidi Stöckl in Women’s Health
