# Supplementary material for: Women’s understanding of economic abuse in North-Western Tanzania
Source: Womens Health (Lond). 2021 Sep 8;17:17455065211042180. doi: 10.1177/17455065211042180 (PMC8436308; doi:10.1177/17455065211042180)
Supplement: sj-docx-2-whe-10.1177_17455065211042180 – Supplemental material for Women’s understanding of economic abuse in North-Western Tanzania [file sj-docx-2-whe-10.1177_17455065211042180.docx]

**Qualitative longitudinal interview guide**

**Main objectives:**

1. To gain a better understanding of how women perceive and define psychological, economic and sexual violence and distinguish it from other behaviour
2. To understand changes and reasons for those changes in the three forms of violence in the relationship
3. To examine how women cope with abuse and violence in their relationship

**Entry to the interview**

Thank you for agreeing to talk to us today. We are very grateful for your long participation in the MAISHA study and that you are willing to share your thoughts further with us. Different to the previous interviews, where we asked you to answer very concrete questions, this time we would like to hear more about your experiences, thoughts and reflections. The MAISHA study is interested in women’s lives and in particular their relationships, the good sides of it and the bad sides of it. Today we would like to hear more about your relationships and how you think about a couples behaviour towards each other, what is good behaviour, what is bad behaviour and what is violence. Our aim is to understand what you consider to be acceptable, non-acceptable and violence and why. Please be assured that there are no right or wrong answer and that we are interested in your opinion. You do not have to answer any of the questions, and you can stop the interview at any time. If you are okay, we will record the conversation.

Are you okay with me to proceed?

**Entry question**

As you know, the MAISHA study has talked a lot about relationships and your relationship with your partner. As we think a lot about relationships, we would like to know from you:

1. What are you looking for in a partner and a relationship? /What are your expectations from a relationship?

   OR: What does a strong/good relationship look like? Do you know a relationship in your neighbourhood/among friends/ or in your family that you think is strong or good?

Probe: What do you like in your partnership? What do you **not** like in your partnership?

**Leading to their own relationship**

We would like to know more about your own relationships, your current ones or past ones.

1. Can you tell me about those relationships with men that were important to you (touched your life)?

   Potential probes: How many serious relationships have you had? Did you live together?
   Would you say they were good or bad relationships?
2. Can you tell us more about your current relationship?

   Potential probes: Can you tell me how you met and became a couple? Do you find in your current relationship what you are looking for in a relationship/partner?

**Leading to their communication and psychological abuse**

1. Can you tell us about your daily communication with your current or last partner?

   Potential probes: How often do you talk? What do you talk about?
2. If she had a previous partner and communication in the current relationship are good, focus on the previous relationship: How was your communication in your previous relationship (you can compare to the current one)?

   If she mentions it is not good in the current/past relationship, PROBE: What is it that you do not like about the communication (the way he talks, body language, no communication?)? Why does this happen? What happens? Can you give examples?

We are interested in understanding what kind of communication in a relationship is good and healthy and what is harmful and abusive.

1. Would you consider any of the talk that you do not like as abusive or even violent?
   OR: Does it happen sometime that he talks or communicates with you in a way that you think is abusive or violence?

   Probe: Is/Was any of his talk hurtful or harmful to you or upsetting you on a regular basis?
   Can you give an example? Can you tell us about it?

   Probe if the following is not mentioned before:
   - Making bad comments about you in front of others that reflect negative on you?
   - Calling you bad words or swear words?
   - Has he ever threatened you to take the children away from you or to harm the children?
   - Not eating or complaining about the food you prepared?
   - Comment badly on your appearance?

   - Are there any other forms of talk and communication that you experienced as harmful/ hurtful/

making you upset on a regular basis?

1. Are there specific circumstances that you think make any of these insults violence, such as body language, presence of others ….
2. How does it make you feel?
3. Do you feel your communication with your partner and (if mentioned before his harmful way of talking to you) changed over the time of your relationship?
   If yes, Probe: How did it change? Why do you think if changed? Is there a specific event?
4. How do you react to this kind of abusive talk? And how does your partner react to that?
5. Is this something you talk to others about?
   If yes: Who, what do they say, how they reacted, when they did it? What happened afterwards? Was it a good or bad experience?
   If no: Why not?

**Economic household decisions - abuse**

1. How do your partner and you deal with your finances?

   Probe: How much control do you have over your finances and your partners finances?
2. What kind of expenses should a husband pay? What kind of expenses should a wife pay?
3. Do you ever argue about money in a relationship?
   Probe If yes, can you give me an example? How often does it happen? Does it lead to conflicts?
   Probe if no: How confident are you to disagree with your partner?

Many women who experience violence say they also experience economic abuse or violence.

1. Do you think there is a kind of behaviour that you would consider to be economic violence that a partner can do to a woman?
   If yes, please give an example.
   If no, why not

If not mentioned before, please probe (if she mentioned before that she experienced this, ask her if her experience of the following was violence):
- What if he takes the money she earned?
- What if he does not share his money although it is needed for school fees or other expenses?
- What if he does not allow the partner to earn money?

1. To any of the above, does it make a difference if a couple is married whether it is violence?
   Probe further: Does it make a difference if they have children together or not?
2. If you argued with your partner over money or you experienced any form of economic abuse did it changed over the time of your relationship, e.g. did it become more or less?
   If yes, Probe: Why do you think if changed? Is there a specific event?
3. Can women talk to others about this kind of economic/financial abuse?
   If she experienced any of this: did you talk to anyone about this?
   If yes, who can they go to? How do people react?
   If not: Why not?

**Sexual relationship – abuse**

The following questions are about sexual relationships between you and your partners. Again we are keen to hear about your experiences and opinions.

1. Could you tell me about the first time you had sex?
   Probe: When did it happen? What happened? How did it happen? Who was he?
   Probe more: how did you feel afterwards? How did he react afterwards? Have you told anyone about it (if yes, how did they react and make you feel?)

1. In your current relationship, how does your partner show you he is interested in sex and how do you show him? Probe: Is it something you talk about with each other? Probe why not or how?
2. What happens if your partner comes home and wants sex and you do not want to?
   What happens?

   Probe: What do you do? What does he do? Under what conditions can you say no? What happens then? Why do you give in? How do you feel afterwards?

Probe: What happens if your partner wants a certain type of sex that you do not want? What happens?

1. In case she did not report any of 3 to have happened ask:
   Is sex something you sometimes or often disagree or argue about?
   If yes, why? Can you give an example? What do you argue about? What happens once you argue?
2. Based on what we have discussed just now, do you think any of this kind of behaviour is violent or abusive?

   If not mentioned before, ask: Do you think any of the following behaviours would be considered sexual violence and why (questions can also be asked instead of ‘you’ using ‘someone you know’):

   - If a partner physically forces you to have sex even though you do not want to
   - If a partners threatens to leave you if you do not have sex wit them even though you or they do not want to?
   - If you have sex with your partner even though you do not want to because you are afraid of saying no?
   - if your partner insists that you have sex even though you have your menstruation?
   - If your partner has another wife or sexual relationship

   If the respondent is unclear, ask: Do you think this behaviour harmful or hurtful?

Probe further: Are there other examples of sexual violence that you or anyone you know experienced in a relationship that we did not discuss?
Probe: or are there examples of behaviour where you would say you are not sure?

1. Did any of the experiences you described to me before happen from the beginning and did they change during the relationship (can refer to other people’s experiences)?

   If no probe: if no: What and how did they changed?
   Why do you think his behaviour changed? Any specific events?
   What do you think are the reasons for this behaviour?
2. How did you/someone you know respond to the experiences you described before?

   Probe: How did you/someone else react immediately afterwards?

Probe: Did you talk to anyone about this?
**If yes probe**: With whom? How did they react? How did the response make her feel?
**If not:** Why not? When do you think a woman is justified to talk about this and seek help?

Who is it you/women in this situation can talk to about this sensitive issue? Probe whether they would go to faith leaders and traditional healers?

**Children – role of children**

1. Moving to a different topic – can you tell me more about your children?
2. How did having children change your relationship with your partner? Probe: did it make you get closer to each other? Did you argue more?
3. Can you tell me how your partner interacts with the children?
   Probe: does he play with them? Does he only provide financially for them? Does he listen to them when they tell him about their day? Does he have favourites? Does he bring them to school or the doctor?
4. How do your children affect the way you react to the abusive behaviour? Can you give an example? Do you think of your children when you think of how to best respond to the abusive behaviours?

**Positive ending**

What advice would you give to young women and young couples based on your experiences in your relationship so that they can make their relationship strong?

Probe: What strategies do you use?
